# Association between fatty acids intake and bone mineral density in adolescents aged 12-19: NHANES 2011–2018

**DOI:** 10.3389/fendo.2024.1402937

**Published:** 2024-07-09

**Authors:** Zhi-Gang Wang, Ze-Bin Fang, Xiao-Li Xie

**Affiliations:** ^1^ Department of Emergency, Beijing University of Chinese Medicine Shenzhen Hospital (Long gang), Shenzhen, China; ^2^ The First School of Clinical Medicine, Guangzhou University of Chinese Medicine, Guangzhou, China

**Keywords:** bone mineral density, saturated fatty acids, monounsaturated fatty acids, polyunsaturated fatty acids, adolescent, NHANES

## Abstract

**Background:**

The relationship between the intake of dietary fatty acids (FA) and bone mineral density (BMD) has been the subject of prior investigations. However, the outcomes of these studies remain contentious. The objective of this research is to examine the link between dietary FA consumption among adolescents and BMD.

**Methods:**

This study utilized high-quality data from the National Health and Nutrition Examination Survey database, spanning 2011 to 2018, to explore the association between dietary fatty acids and bone health indicators in adolescents, including BMD and bone mineral content (BMC). Analyses were performed using weighted multivariate linear regression models, incorporating detailed subgroup analysis.

**Results:**

The study included 3440 participants. Analysis demonstrated that intake of saturated fatty acids (SFA) was positively correlated with total BMD, left arm BMD, total BMC, and left arm BMC. Monounsaturated fatty acid (MUFA) intake was positively correlated with BMC across most body parts, though it showed no correlation with BMD. Intake of polyunsaturated fatty acids (PUFA) was significantly inversely correlated with both BMD and BMC in most body parts. Additionally, subgroup analysis indicated that variables such as sex, age, standing height, and race significantly influenced the correlation between FA intake and BMD.

**Conclusions:**

Our study indicates that dietary intake of SFA may benefit to BMD in adolescents, in contrast to PUFA and MUFA. Therefore, we recommend that adolescents maintain a balanced intake of SFA to promote optimal bone mass development while preserving metabolic health.

## 1 Introduction

Osteoporosis (OP), a systemic disease affecting the musculoskeletal framework, is characterized by reduced bone density, degeneration of bone tissue structure, increased susceptibility to fractures, and enhanced fragility (1, 2). Given the aging population and increased life expectancy, the World Health Organization has recognized OP as one of the most pressing global public health issues. In recent years, there has been noticeable increase in the incidence of OP. A study by the International Society for Clinical Densitometry and the International Osteoporosis Foundation projects that by 2030, osteoporosis will affect over 70 million individuals in America, a condition marked by decreased bone mineral density (BMD) (3). Statistics indicate that osteoporosis-related fractures result in an estimated annual direct economic loss of 17 billion US dollars worldwide, posing a significant economic burden on healthcare systems across various countries (4). Currently, the clinical diagnosis and assessment of OP rely on BMD measurements, a method proven reliable and effective in numerous studies (5–7). Consequently, the early detection, intervention, and management of OP have attracted increasing interest among researchers.

In recent years, adolescent dietary patterns in economically developed countries have increasingly shifted towards processed and calorie-dense foods (8). From 2009 to 2019, there was a significant increase in the proportion of U.S. teenagers-across all genders and racial demographics-consuming fruit or 100% juice less than once daily (9). Similarly, the daily vegetable consumption among teenagers has notably declined. Insufficient intake of fruits and vegetables correlates with deficiencies in vital nutrients essential for bone health and development. Specifically, essential nutrients for bone health, such as calcium, vitamin D, and protein, are primarily derived from dairy products, green leafy vegetables, and other nutrient-rich sources. Inadequate intake of calcium and vitamin D is linked to lower bone density and a heightened fracture risk among adolescents (10). Furthermore, while protein supports bone growth, excessive intake can adversely affect bone health, particularly if not balanced with adequate calcium (10). Furthermore, a plethora of other nutrients are integral to bone health. Essential trace elements such as zinc, copper, manganese, and boron, in conjunction with critical vitamins like vitamin K and vitamin C, significantly influence bone structure and integrity (11, 12). Emerging research further delineates dietary fat as an instrumental regulatory element in the preservation of musculoskeletal structure and functionality (13–18). Fatty acids (FA) have garnered increasing attention due to their significant significance as a crucial constituent of dietary fat and their potential regulatory role in metabolic disorders. FA are classified into three distinct groups based on the saturation level of their hydrocarbon chains: saturated fatty acids (SFA), which contain no double bonds; monounsaturated fatty acids (MUFA), characterized by the presence of a single double bond; and polyunsaturated fatty acids (PUFA), which possess multiple double bonds (16). This categorization reflects the structural differences and physiochemical properties attributable to the degree of saturation within the hydrocarbon chains of fatty acids.

Recent research into dietary fatty acids’ influence on bone mineral density presents varied and often contradictory outcomes (19–23). Such discrepancies likely arise from small sample sizes, varied survey methodologies, and inherent selection biases. Alarmingly, few studies have assessed fatty acids’ effects on bone health in adolescents. Given that adolescence is a crucial period for bone development, ensuring optimal nutrition is imperative to foster peak bone density and quality, which are essential for long-term health. We hypothesize that dietary FA intake is associated with BMD in adolescents; however, this association is likely non-linear and modulated by variables including age, gender, and ethnicity. Thus, it is vital to further investigate the link between fatty acids and bone health during adolescence. This study utilizes data from the National Health and Nutrition Examination Survey (NHANES) to delve into how dietary fatty acids influence adolescent bone health and to develop novel clinical intervention strategies.

## 2 Methods

### 2.1 Data source and study population

NHANES is designed to assess the health and nutritional status of the American population across a broad age spectrum. The study stands out due to its distinctive integration of questionnaires and physical evaluations. The survey is administered on an annual schedule, utilizing a sample that is representative of the entire nation and consisting of roughly 5,000 individuals. These individuals are situated in various counties throughout the whole country, performing visits to a total of 15 counties annually. The NHANES interviews encompassed a range of inquiries about demographics, socioeconomic status, dietary habits, and health-related factors. Skilled medical professionals undertake a battery of diagnostic tests, including physical, dental, and physiological evaluations, as well as laboratory analysis. The primary purpose of utilizing the data collected from this survey is to facilitate epidemiological and health science research. The objective of NHANES is to support the development of comprehensive public health policies and promote health education across the broader population (24).

**Figure 1:**
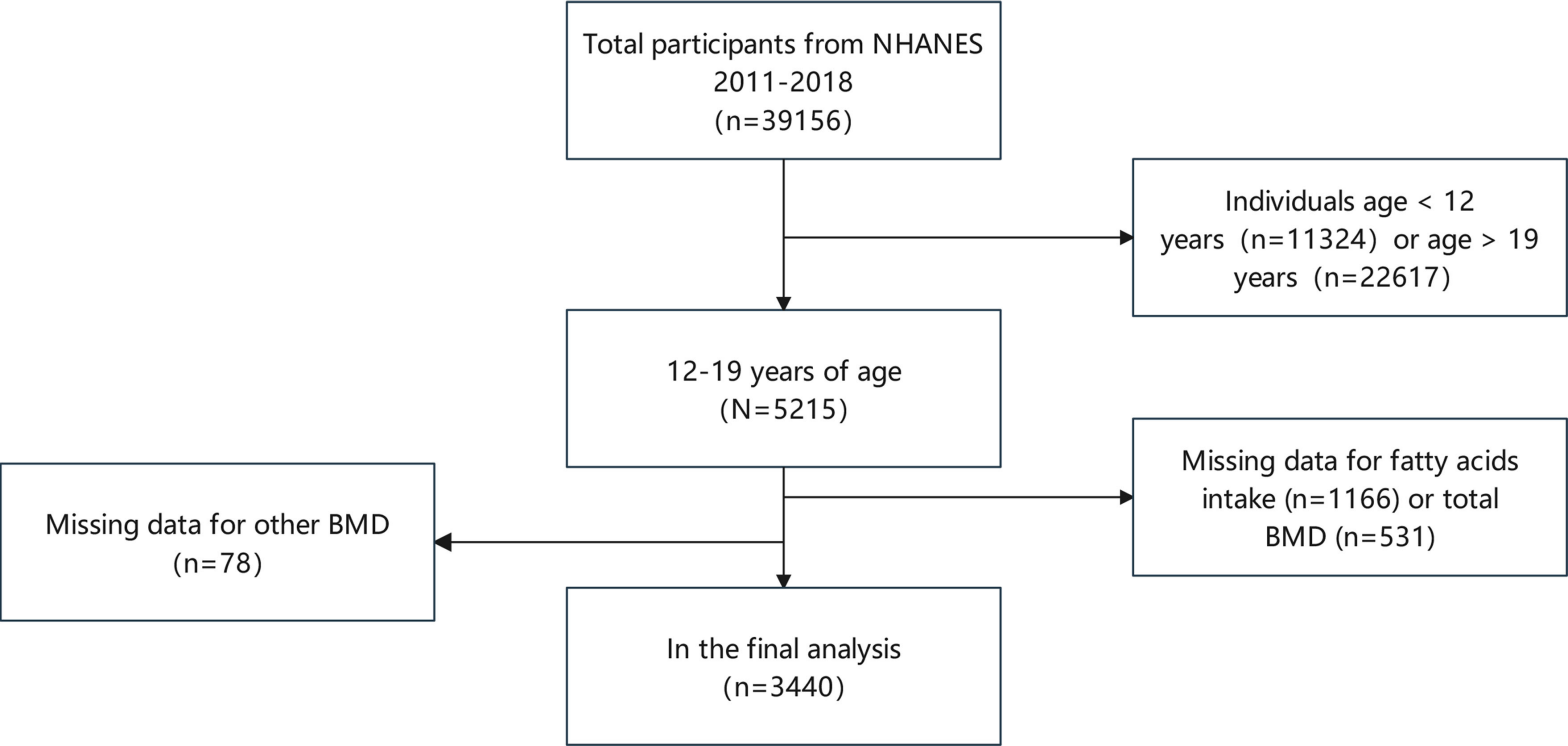
Participant selection flow chart. NHANES, National Health and Nutrition Examination Survey; BMD, bone mineral density.

Our cross-sectional study examined 39,156 NHENAS participants from 2011 to 2018. This study examined the correlation between dietary fatty acids and adolescent bone health, focusing on BMD and BMC in individuals aged 12-19. Recognizing that regional bone properties may be influenced by distinct factors, we conducted a comprehensive analysis that incorporated BMD and BMC measurements from various anatomical sites and assessed multiple classes of FA. Consequently, participants under 12 years of age (11,324 individuals) and those older than 19 years (22,617 individuals) were excluded. Additionally, individuals lacking fatty acid intake data (1,166 participants), total BMD measurements (531 participants), or other BMD data (78 participants) were also excluded. Following the completion of this screening process, a cumulative total of 3440 people were deemed eligible for inclusion in the study (**Figure 1**).

### 2.2 Ethics statement

Prior to their involvement in the survey, participants received detailed explanations about the nature and specifics of the study, following which they executed a consent agreement. This process of informed consent received approval from the National Center for Health Statistics’ Ethics Review Board. After the process of formal anonymization is concluded, the entirety of the data is released to the public to optimize the utilization of these resources. The accessibility of these statistics is contingent upon adherence to the NHANES database restrictions and a commitment to statistical analysis. All experimental research conducted using this data must adhere to the relevant laws and legislation.

### 2.3 Covariates

Daily FAs consumption was the independent variable in this study. All participants in the NHANES underwent two 24-hour food recall interviews, both of which were administered by proficient dietary interviewers who were fluent in both Spanish and English. The initial in-person interviews took place within designated private rooms at the Mobile Examination Centre (MEC), wherein a standardized collection of measuring guides was utilized. The subsequent 24-hour dietary recall interview is conducted via telephone, often within a time frame of 3 to 10 days following the MEC diet assessment. Furthermore, this study used categorical variables, including gender, ethnicity, and moderate physical activity. Continuous variables include the ratio of family income to poverty, body mass index (BMI), standing height, alkaline phosphatase (ALP), serum calcium (Ca), serum phosphorus (P), serum uric acid (UA), total cholesterol (TC), triglycerides (TG), glycohemoglobin, blood urea nitrogen (BUN), serum creatinine (Scr), urinary albumin-creatinine ratio (UACR), total protein (TP), vitamin D (VitD) intake, alcohol intake, energy intake, carbohydrate intake, protein intake, cholesterol intake, as well as BMD (lumbar spine, left arm, left leg, head, trunk, thoracic, pelvis, and left rib), and BMC (total, lumbar spine, left arm, left leg, head, thoracic, trunk, pelvis, and left rib). At www.cdc.gov/nchs/nhanes/, you can find out more about the collection of covariates and the 24-hour dietary recall interview.

### 2.4 Outcome variable

Dual-energy X-ray absorptiometry (DXA) is a highly prevalent technique for assessing body composition due to its rapidity, user-friendliness, and minimal radiation exposure (25). DXA detection results are often used for osteoporotic fractures, fracture risk prediction, and drug efficacy evaluation. The Hologic Discovery A is a bone densitometer that utilizes fan-beam X-ray technology. Manufactured by Hologic, Inc. in Bedford, Massachusetts, this device uses an energy tube to generate two distinct energy levels. These energy levels are then used to determine BMC and BMD. All DXA scans are performed by a certified radiographer. Additional information regarding the operational mechanisms of the DXA examination can be found on the official website of the NHANES, which offers a comprehensive body composition manual.

### 2.5 Statistical analysis

Statistical analyses were conducted using EmpowerStats2 (http://www.empowerstats.com) and R software (version 3.4.4), considering *P*-values below 0.05 as statistically significant. Sample sizes were weighted. Continuous variables were described as mean ± standard deviation, and categorical variables as percentages for baseline comparison. *P*-values for continuous and categorical variables were derived using weighted linear regression and chi-square tests, respectively. Furthermore, weighted multiple regression models assessed the association between dietary FA intake and BMD metrics (total, lumbar spine, and left arm), with adjustments for covariates outlined in **Table 1**. Linear trend tests were employed to analyze effect size trends. To enhance data utilization, subgroup analyses were stratified by gender, age, standing height, and race, enriching our insights into the relationships between FA intake and BMD.

**Table 1:** Weighted characteristics of the study sample.

Quartiles of total bone mineral density (g/cm2)	Lowest quartiles	2nd	3rd	4th	*P* value
**Age (years)**	13.53 ± 1.77	15.26 ± 2.13	16.12 ± 1.96	17.11 ± 1.60	< 0.001
**Gender (%)**					< 0.001
male	53.14	43.53	44.74	66.67	
female	46.86	56.47	55.26	33.33	
**Race/ethnicity (%)**					< 0.001
White people	55.47	57.85	53.54	43.13	
Black people	6.27	10.08	14.57	24.44	
Mexican American	17.96	13.75	15.29	18.05	
Other race	20.3	18.33	16.59	14.38	
**Ratio of family income to poverty (%)**	2.52 ± 1.58	2.45 ± 1.58	2.31 ± 1.57	2.29 ± 1.60	0.006
**Body mass index (kg/m2)**	21.29 ± 4.94	22.95 ± 5.31	25.24 ± 6.13	26.57 ± 6.34	< 0.001
**Standing height(cm)**	158.67 ± 8.33	164.62 ± 8.23	167.38 ± 8.79	171.52 ± 8.39	< 0.001
**Moderate activities (%)**					< 0.001
No	29.1	25.52	25.39	28.54	
Yes	50.43	57.09	57.83	60.78	
Don’t know	20.48	17.39	16.78	10.69	
**Alkaline phosphatase (u/L)**	201.34 ± 102.64	138.16 ± 87.04	107.94 ± 62.79	91.19 ± 39.60	< 0.001
**Serum calcium (mmol/L)**	2.41 ± 0.08	2.40 ± 0.07	2.39 ± 0.07	2.40 ± 0.07	< 0.001
**Serum phosphorus (mmol/L)**	1.52 ± 0.19	1.40 ± 0.21	1.36 ± 0.18	1.32 ± 0.16	< 0.001
**Serum uric acid (umol/L)**	283.70 ± 61.65	294.17 ± 67.50	303.01 ± 71.78	322.51 ± 70.76	< 0.001
**Total cholesterol (mmol/L)**	4.07 ± 0.71	4.08 ± 0.71	4.04 ± 0.76	4.08 ± 0.75	0.582
**Triglycerides (mmol/L)**	1.11 ± 0.65	1.12 ± 0.72	1.16 ± 0.76	1.12 ± 0.71	0.561
**Glycohemoglobin (%)**	5.27 ± 0.41	5.25 ± 0.44	5.24 ± 0.31	5.23 ± 0.31	0.093
**Blood urea nitrogen (mmol/L)**	3.99 ± 1.21	3.90 ± 1.13	4.12 ± 1.20	4.22 ± 1.13	< 0.001
**Serum creatinine (umol/L)**	54.44 ± 11.41	61.34 ± 11.47	66.04 ± 12.54	74.46 ± 13.08	< 0.001
**Urinary albumin creatinine ratio (mg/g)**	35.13 ± 124.55	24.06 ± 85.26	22.06 ± 87.35	14.87 ± 38.19	< 0.001
**Total protein (g/L)**	72.07 ± 3.98	72.58 ± 3.83	72.68 ± 3.87	72.69 ± 4.06	0.003
**Vitamin D intake (mcg/d)**	5.39 ± 4.67	4.50 ± 3.60	4.62 ± 4.12	5.18 ± 4.70	< 0.001
**Alcohol intake (g/d)**	0.07 ± 1.05	0.49 ± 3.75	0.63 ± 5.56	1.28 ± 6.58	< 0.001
**Energy intake (kcal/d)**	1921.21 ± 655.70	1868.20 ± 704.25	1915.88 ± 744.43	2207.70 ± 854.88	< 0.001
**Carbohydrate intake (g/d)**	248.87 ± 91.16	243.11 ± 88.67	242.51 ± 95.98	274.64 ± 110.90	< 0.001
**Protein intake (g/d)**	71.54 ± 27.98	69.57 ± 34.39	74.46 ± 40.84	86.33 ± 41.35	< 0.001
**Cholesterol intake (mg/d)**	224.37 ± 139.31	222.59 ± 167.22	246.20 ± 201.71	285.22 ± 186.41	< 0.001
**Total saturated fatty acids intake (g/d)**	25.67 ± 11.83	23.72 ± 11.59	25.01 ± 12.61	29.10 ± 14.04	< 0.001
**Total monounsaturated fatty acids intake (g/d)**	24.74 ± 10.60	23.83 ± 11.66	24.94 ± 12.21	29.79 ± 15.09	< 0.001
**Total Polyunsaturated fatty acids intake (g/d)**	16.26 ± 7.80	16.70 ± 9.04	16.85 ± 9.17	19.36 ± 9.94	< 0.001
**Lumbar Spine Bone Mineral Density (g/cm2)**	0.80 ± 0.11	0.94 ± 0.09	1.03 ± 0.10	1.12 ± 0.12	< 0.001
**Left Arm Bone Mineral Density (g/cm**2)	0.61 ± 0.05	0.68 ± 0.04	0.73 ± 0.05	0.81 ± 0.07	< 0.001
**Left Leg Bone Mineral Density (g/cm**2)	0.94 ± 0.08	1.05 ± 0.06	1.14 ± 0.06	1.27 ± 0.10	< 0.001
**Head Bone Mineral Density (g/cm2)**	1.48 ± 0.19	1.75 ± 0.20	1.94 ± 0.24	2.16 ± 0.26	< 0.001
**Trunk Bone Mineral Density (g/cm2)**	0.71 ± 0.06	0.82 ± 0.04	0.90 ± 0.05	1.01 ± 0.08	< 0.001
**Thoracic Bone Mineral Density (g/cm**2)	0.62 ± 0.07	0.72 ± 0.05	0.78 ± 0.06	0.86 ± 0.08	< 0.001
**Pelvis Bone Mineral Density (g/cm**2)	0.99 ± 0.11	1.13 ± 0.09	1.24 ± 0.10	1.38 ± 0.14	<0.001
**Left Rib Bone Mineral Density (g/cm**2)	0.53 ± 0.05	0.60 ± 0.04	0.64 ± 0.04	0.72 ± 0.07	< 0.001
**Total Bone Mineral Content(g)**	1507.12 ± 233.25	1887.45 ± 202.25	2179.48 ± 236.61	2616.33 ± 332.20	< 0.001
**Lumbar Spine Bone Mineral Content (g)**	35.52 ± 9.43	48.38 ± 8.84	54.47 ± 9.20	63.56 ± 11.37	< 0.001
**Left Arm Bone Mineral Content (g)**	99.08 ± 21.40	130.23 ± 22.98	153.10 ± 27.52	189.37 ± 35.78	< 0.001
**Left Leg Bone Mineral Content (g)**	295.04 ± 55.00	360.79 ± 56.08	413.12 ± 65.78	500.52 ± 83.86	< 0.001
**Head Bone Mineral Content (g)**	327.88 ± 46.81	393.24 ± 48.31	442.50 ± 56.81	509.54 ± 69.27	< 0.001
**Thoracic Bone Mineral Content (g)**	69.77 ± 17.62	91.58 ± 16.45	105.66 ± 18.52	119.29 ± 20.12	< 0.001
**Trunk Bone Mineral Content (g)**	382.05 ± 76.76	500.39 ± 67.96	589.33 ± 79.43	709.34 ± 107.80	< 0.001
**Pelvis Bone Mineral Content (g)**	163.23 ± 38.08	220.72 ± 38.06	270.03 ± 45.82	340.38 ± 68.99	< 0.001
**Left Rib Bone Mineral Content (g)**	57.59 ± 12.30	70.86 ± 11.31	80.58 ± 13.52	94.72 ± 15.93	< 0.001

Continuous variables are presented as Mean ± SD, P-value was calculated by a weighted linear regression model. Categorical variables are presented as %, P-value was calculated by the chi-square test.

## 3 Results

### 3.1 Characteristics of participants

**Table 1** displays the weighted sociodemographic and physiological characteristics of the participants. Following stratification of total BMD into quartiles, we observed significant differences across multiple variables: age, gender, race, the ratio of family income to poverty, BMI, standing height, and moderate activity. Biochemical parameters such as ALP, Ca, P, UA, BUN, Scr, UACR, TP also varied significantly. Additionally, intakes of VitD, alcohol, energy, carbohydrate, protein, cholesterol, total SFA, total MUFA, total PUFA were distinct among the quartiles. BMD at various sites including lumbar spine, left arm, left leg, head, trunk, thoracic, pelvis, left rib, along with bone mineral content (BMC) at these locations, showed significant variances. In contrast, no significant differences were observed in glycohemoglobin, TC, and TG. These findings suggest that BMD is potentially influenced by demographic characteristics, dietary habits, and physical activity levels.

### 3.2 Association between total SFAs intake, BMD, and BMC

**Figure 2:**
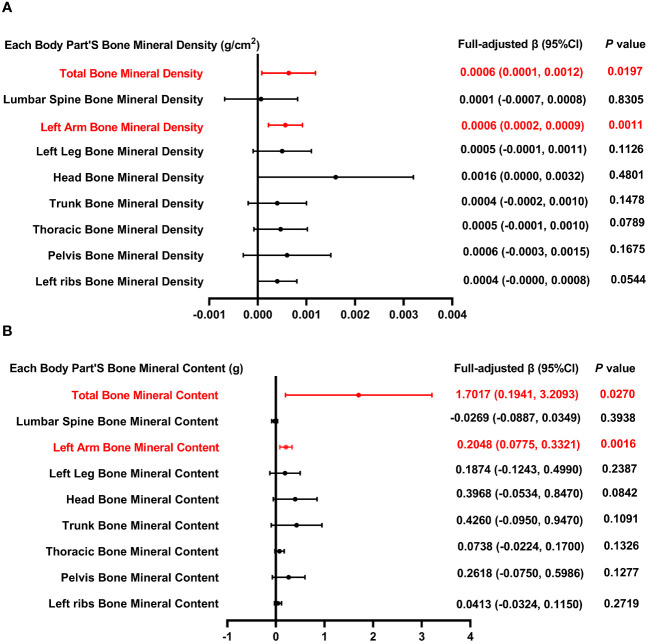
Forest plot of the relationship between total SFA intake and BMD and BMC for each body part. **(A)** Correlation between BMD and total SFA intake in each body part. **(B)** Correlation between BMC and total SFA intake in each body part. SFA, saturated fatty acids intake; BMD, bone mineral density; BMC, bone mineral content.

**Figure 2** illustrates the association between total SFA intake and both BMD (**Figure 2A**) and BMC (**Figure 2B**). Positive correlations were observed between the overall intake of SFA and both total and left arm BMD, with *P*-values of 0.0197 and 0.0011, respectively. Similarly, positive correlations persisted for both total and left arm BMC, with *P*-values of 0.0270 and 0.0016, respectively. Three weighted multivariate linear regression models of total SFA intake versus total BMD, lumbar spine BMD, and left arm BMD are shown in **Table 2**. Adjustments were made for all factors. The stratification variable was not adjusted for in the subgroup analysis.

**Table 2:** Association between total saturated fatty acids intake (g/d) and bone mineral density (g/cm^2^).

Exposure	Total BMD β, 95%Cl, *P* value	Lumbar Spine BMD β, 95%Cl, *P* value	Left Arm BMD β, 95%Cl, *P* value
Quartiles of total polyunsaturated fatty acids intake (g/d)			
Lowest quartiles (2.00-16.29)	reference	reference	reference
2nd (16.30-23.24)	-0.0079 (-0.0163, 0.0004)	-0.0126 (-0.0242, -0.0011)*	-0.0101 (-0.0156, -0.0046)***
3rd (23.25-31.64)	0.0048 (-0.0048, 0.0145)	0.0043 (-0.0091, 0.0177)	-0.0025 (-0.0088, 0.0038)
4th (31.65-116.23)	0.0170 (0.0035, 0.0305)*	0.0073 (-0.0114, 0.0260)	0.0031 (-0.0057, 0.0120)
P *for* trend	0.007	0.172	0.341
Stratified by gender			
Male	0.0009 (0.0002, 0.0016)*	0.0004 (-0.0005, 0.0014)	0.0007 (0.0002, 0.0012)**
Female	0.0003 (-0.0006, 0.0011)	-0.0007 (-0.0019, 0.0006)	0.0005 (-0.0000, 0.0009)
Stratified by age (years old)			
12	0.0013 (-0.0002, 0.0028)	0.0002 (-0.0018, 0.0023)	0.0007 (-0.0003, 0.0017)
13	0.0014 (-0.0002, 0.0029)	0.0029 (0.0008, 0.0050) **	0.0008 (-0.0003, 0.0018)
14	-0.0004 (-0.0019, 0.0011)	-0.0007 (-0.0026, 0.0012)	0.0004 (-0.0005, 0.0013)
15	0.0014 (-0.0004, 0.0032)	0.0013 (-0.0012, 0.0037)	0.0005 (-0.0007, 0.0016)
16	0.0007 (-0.0008, 0.0022)	-0.0007 (-0.0028, 0.0015)	0.0002 (-0.0008, 0.0012)
17	0.0010 (-0.0006, 0.0026)	0.0010 (-0.0013, 0.0034)	0.0014 (0.0005, 0.0024) **
18	-0.0007 (-0.0024, 0.0009)	-0.0007 (-0.0030, 0.0015)	0.0003 (-0.0008, 0.0013)
19	0.0011 (-0.0004, 0.0027)	0.0012 (-0.0010, 0.0034)	0.0008 (-0.0003, 0.0019)
Stratified by standing height (cm)			
Q1 (132.9-160.2)	0.0004 (-0.0006, 0.0014)	0.0002 (-0.0013, 0.0017)	0.0001 (-0.0005, 0.0008)
Q2 (160.3-168.7)	0.0007 (-0.0003, 0.0017)	-0.0001 (-0.0014, 0.0013)	0.0007 (0.0001, 0.0013)*
Q3 (168.8-190.9)	0.0010 (0.0001, 0.0019)*	0.0004 (-0.0009, 0.0016)	0.0010 (0.0004, 0.0016)**
Stratified by race			
White people	0.0009 (-0.0001, 0.0019)	-0.0003 (-0.0017, 0.0011)	0.0008 (0.0002, 0.0015)*
Black people	0.0003 (-0.0010, 0.0015)	-0.0001 (-0.0019, 0.0017)	0.0005 (-0.0003, 0.0012)
Mexican American	0.0016 (0.0002, 0.0029)*	0.0023 (0.0006, 0.0041)**	0.0006 (-0.0003, 0.0015)
Other race	-0.0010 (-0.0020, 0.0001)	-0.0008 (-0.0023, 0.0006)	-0.0002 (-0.0009, 0.0005)

All factors were adjusted. In the subgroup analysis, not adjusted for the stratification variable itself. *P < 0.05, **P < 0.01, ***P < 0.001.

The lowest quartiles of total SFA were used as a control group in the weighted multiple linear regression model of total BMD and total SFA intake. In the highest quartile of total SFA, a positive association with total BMD was identified, exhibiting statistical significance (*P* < 0.05), with the trend’s *P* value also reaching a notable level of significance (*P* = 0.007). In the process of performing subgroup analysis based on gender, age, and race, it was shown that in male and Mexican Americans, there existed a positive association between total SFA intake and total BMD (*P* < 0.05). We also stratified the standing height, with 132.9-160.2 cm as Q1, 160.3-168.7 cm as Q2, and 168.8-190.9 cm as Q3, and only in Q3, there was a positive association between total SFA intake and total BMD. This phenomenon could be attributed to the generally larger bone size and greater bone mass observed in men, with testosterone significantly contributing to the preservation of bone density. Moreover, taller individuals typically exhibit elevated levels of growth and sex hormones, which are associated with enhanced bone growth and density. These hormonal effects may be further influenced by biomechanical stimulation, genetic factors, among other determinants. Furthermore, dietary preferences among Mexicans, which often include high-calcium foods, might also play a role in influencing bone density.

In the model of lumbar spine BMD and total SFA intake, when SFA intake was stratified by quartiles, with the lowest quartiles as the reference, the 2nd quartile of total SFA was negatively correlated with total BMD (*P* < 0.05). Nonetheless, the trend across quartiles did not reach statistical significance (*P* = 0.172). Further subgroup analysis, which was categorized by gender, age, standing height, and race, revealed a significant positive correlation between dietary SFA and lumbar spine BMD exclusively in adolescents aged 13 and in Mexican American individuals (*P* < 0.01). After accounting for confounding factors, the overall correlation between SFA intake and BMD may be obscured (**Figure 2**). However, this correlation might become significant when analyses are conducted separately for particular subgroups, suggesting unique relationships within these populations.

The measurement of BMD in the left arm provides valuable insights for the young population, particularly in evaluating overall bone health and estimating the risk of fractures. As illustrated in **Figure 2**, a positive correlation exists between dietary SFA intake and BMD. In the analysis concerning the relationship between left arm BMD and overall SFA consumption, segmentation of SFA intake into quartiles revealed a negative association between the second quartile of SFA intake and left arm BMD (*P* < 0.001). Nonetheless, the trend across quartiles did not achieve statistical significance (*P* = 0.341). In the subgroup analysis stratified by gender, age, and race, a significant positive association between dietary SFA and BMD in the left arm was observed exclusively in males, 17-year-olds, and white people. In subgroups stratified by standing height, dietary SFA intake was positively related to left arm BMD in Q2 and Q3, with significance levels of *P* < 0.05 and *P* < 0.01, respectively.

### 3.3 Association between total MUFA intake, BMD, and BMC

**Figure 3:**
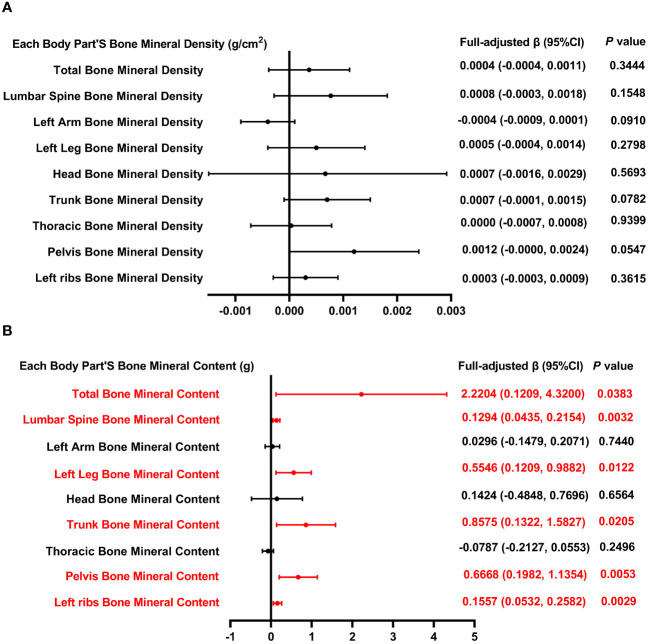
Forest plot of the relationship between total MUFA intake and BMD and BMC for each body part. **(A)** Correlation between BMD and total SFA intake in each body part. **(B)** Correlation between BMC and total SFA intake in each body part. MUFA, monounsaturated fatty acids intake; BMD, bone mineral density; BMC, bone mineral content.

**Figure 3** illustrates the correlation between total MUFA and both BMD and BMC. **Figure 3B** illustrates that BMC measurements across multiple anatomical regions—including the total body, lumbar spine, left leg, trunk, pelvis, and left ribs—demonstrate a positive relationship with overall MUFA intake. However, the association between MUFA intake and BMD across these regions did not reach statistical significance (*P >* 0.05).

**Table 3** outlines three models that examine the relationship between MUFA consumption and BMD, with adjustments made for all pertinent variables. The analysis of total BMD in relation to MUFA intake, categorized by quartiles, reveals a negative correlation in the 2nd quartile with total BMD (*P* < 0.01), without a significant trend across quartiles (*P* = 0.850). Furthermore, when dissecting the data by gender, age, standing height, and race, a significant negative relationship between total MUFA intake and total BMD was observed exclusively in the “other race” category (*P* < 0.05). In the model of lumbar spine BMD and total dietary MUFA. When categorized by gender, age, standing height, and race, positive correlations between dietary MUFA and lumbar spine BMD and MUFA were found only in female, 16-year-olds, and white people (*P* < 0.05); however, in 13-year-olds, lumbar spine BMD was negatively correlated with total MUFA intake. In the analysis of the left arm BMD model, stratification of MUFA across quartiles revealed that MUFA levels within the second and third quartiles exhibited a significant inverse relationship with BMD, as evidenced by *P*-values of <0.001 and <0.01, respectively; the *P* for trend was 0.034. When analyses were stratified by gender, age, standing height, and race, total MUFA intake and left arm BMD were negatively associated only in subgroups of 12 and 17 years, Q1, and other race.

**Table 3:** Association between total monounsaturated fatty acids intake (g/d) and bone mineral density (g/cm^2^).

Exposure	Total BMD β, 95%Cl, *P* value	Lumbar Spine BMD β, 95%Cl, *P* value	Left Arm BMD β, 95%Cl, *P* value
Quartiles of total monounsaturated fatty acids intake (g/d)			
Lowest quartiles (2.44-16.79)	reference	reference	reference
2nd (16.80-23.49)	-0.0132 (-0.0217, -0.0047)**	-0.0110 (-0.0228, 0.0008)	-0.0121 (-0.0176, -0.0065)***
3rd (23.491-32.06)	-0.0065 (-0.0164, 0.0035)	0.0021 (-0.0116, 0.0158)	-0.0100 (-0.0165, -0.0035)**
4th (32.062-118.49)	0.0042 (-0.0104, 0.0187)	0.0053 (-0.0149, 0.0254)	-0.0095 (-0.0190, 0.0000)
*P* for trend	0.850	0.412	0.034
Stratified by gender			
Male	0.0003 (-0.0007, 0.0012)	0.0005 (-0.0008, 0.0018)	-0.0003 (-0.0010, 0.0004)
Female	0.0010 (-0.0002, 0.0022)	0.0019 (0.0001, 0.0037)*	-0.0006 (-0.0013, 0.0001)
Stratified by age (years old)			
12	-0.0014 (-0.0034, 0.0007)	0.0016 (-0.0012, 0.0045)	-0.0014 (-0.0027, -0.0001)*
13	-0.0018 (-0.0038, 0.0003)	-0.0035 (-0.0064, -0.0006)*	-0.0010 (-0.0025, 0.0005)
14	0.0002 (-0.0022, 0.0026)	-0.0001 (-0.0031, 0.0029)	-0.0013 (-0.0027, 0.0001)
15	0.0011 (-0.0015, 0.0036)	0.0015 (-0.0020, 0.0050)	0.0008 (-0.0009, 0.0024)
16	0.0006 (-0.0019, 0.0031)	0.0040 (0.0005, 0.0074)*	0.0003 (-0.0014, 0.0019)
17	-0.0005 (-0.0028, 0.0018)	-0.0034 (-0.0068, 0.0000)	-0.0019 (-0.0033, -0.0005)**
18	0.0007 (-0.0012, 0.0025)	-0.0002 (-0.0028, 0.0023)	-0.0004 (-0.0015, 0.0008)
19	0.0019 (-0.0009, 0.0048)	0.0014 (-0.0027, 0.0054)	0.0003 (-0.0016, 0.0023)
Stratified by standing height (cm)			
Q1 (132.9-160.2)	-0.0010 (-0.0024, 0.0005)	0.0008 (-0.0014, 0.0030)	-0.0015 (-0.0025, -0.0006)**
Q2 (160.3-168.7)	0.0000 (-0.0015, 0.0015)	-0.0003 (-0.0023, 0.0016)	-0.0009 (-0.0018, 0.0000)
Q3 (168.8-190.9)	0.0004 (-0.0007, 0.0016)	0.0007 (-0.0009, 0.0023)	-0.0004 (-0.0012, 0.0004)
Stratified by race			
White people	0.0011 (-0.0002, 0.0024)	0.0021 (0.0002, 0.0039)*	-0.0001 (-0.0009, 0.0008)
Black people	-0.0016 (-0.0037, 0.0005)	-0.0026 (-0.0056, 0.0003)	-0.0012 (-0.0024, 0.0001)
Mexican American	0.0012 (-0.0008, 0.0031)	0.0010 (-0.0015, 0.0034)	0.0003 (-0.0009, 0.0015)
Other race	-0.0020 (-0.0035, -0.0005)*	-0.0020 (-0.0040, 0.0000)	-0.0019 (-0.0029, -0.0009)***

All factors were adjusted. In the subgroup analysis, not adjusted for the stratification variable itself. *P < 0.05, **P < 0.01, ***P < 0.001.

### 3.4 Association between total PUFA intake, BMD, and BMC

**Figure 4:**
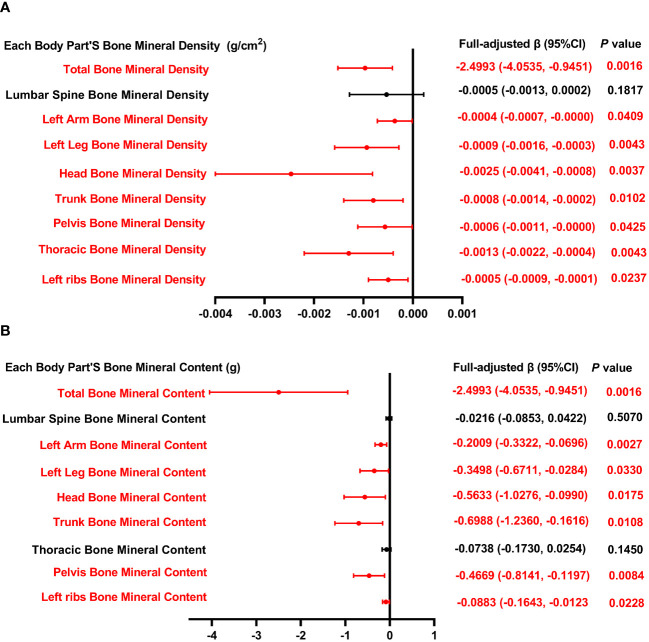
Forest plot of the relationship between total PUFA intake and BMD and BMC for each body part. **(A)** Correlation between BMD and total SFA intake in each body part. **(B)** Correlation between BMC and total SFA intake in each body part. PUFA, monounsaturated fatty acids intake; BMD, bone mineral density; BMC, bone mineral content.

**Figure 4** presents the association between the intake of dietary PUFA and each body part’s BMD as well as BMC. The analysis reveals a consistent inverse relationship across various BMD parameters, including total BMD, BMD of the left arm, left leg, head, trunk, pelvis, thoracic region, and left rib, with the intake of total PUFA. Similarly, this inverse association extends to BMC measurements, encompassing total BMC, BMC of the left arm, left leg, head, trunk, pelvis, and left rib, further supporting the negative association between dietary polyunsaturated fatty acid intake and bone health.

In **Table 4**, we investigate the relationship between overall PUFA consumption and BMD at various anatomical locations. This analysis examines the link between PUFA consumption and BMD, highlighting the influence of factors including gender, age, height, and ethnicity. Specifically, in the context of PUFA consumption and total BMD, a negative correlation is observed among males aged 15 and 19, Q3, and within white people and Mexican American cohorts. Conversely, a significant positive correlation is noted across other ethnic groups. This nuanced examination underscores the complex interplay between dietary PUFA and bone health, contingent upon demographic factors. In models of PUFA and lumbar spine BMD, when PUFA intake was divided into quartiles, there was a positive correlation in the 2nd quartile (*P* <0.05), and the *P* for trend was 0.376. In subgroup analysis, PUFA was significantly inversely associated with lumbar spine BMD only in males, 15-year-olds, and Mexican Americans (*P* <0.05). In the analysis of total PUFA intake and left arm BMD, dividing PUFA intake into quartiles did not reveal a statistically significant trend (*P* = 0.426). Stratifying by gender, age, height, and race, PUFA intake showed a negative correlation with left arm BMD in male, 17-year-olds, Q3, and white people, whereas it exhibited a positive correlation in individuals of other races. The above findings indicate that PUFA could potentially influence BMD negatively.

**Table 4:** Association between total polyunsaturated fatty acids intake (g/d) and bone mineral density (g/cm^2^).

Exposure	Total BMD β, 95%Cl, *P* value	Lumbar Spine BMD β, 95%Cl, *P* value	Left Arm BMD β, 95%Cl, *P* value
Quartiles of total polyunsaturated fatty acids intake (g/d)			
Lowest quartiles (1.29-10.66)	reference	reference	reference
2nd (10.669-15.516)	0.0057 (-0.0024, 0.0139)	0.0135 (0.0023, 0.0247)*	0.0023 (-0.0030, 0.0076)
3rd (15.519-24.66)	-0.0044 (-0.0134, 0.0046)	0.0090 (-0.0034, 0.0215)	-0.0015 (-0.0074, 0.0044)
4th (24.96-51.87)	-0.0073 (-0.0189, 0.0043)	0.0091 (-0.0069, 0.0252)	-0.0017 (-0.0093, 0.0059)
*P* for trend	0.076	0.376	0.426
Stratified by gender			
Male	-0.0012 (-0.0019, -0.0004)**	-0.0010 (-0.0019, -0.0000)*	-0.0005 (-0.0010, -0.0000)*
Female	-0.0008 (-0.0017, 0.0000)	0.0001 (-0.0012, 0.0014)	-0.0003 (-0.0008, 0.0002)
Stratified by age (years old)			
12	-0.0013 (-0.0028, 0.0003)	-0.0012 (-0.0034, 0.0010)	-0.0003 (-0.0013, 0.0008)
13	0.0008 (-0.0007, 0.0024)	0.0006 (-0.0016, 0.0028)	0.0005 (-0.0006, 0.0016)
14	0.0001 (-0.0013, 0.0015)	0.0005 (-0.0012, 0.0023)	-0.0000 (-0.0009, 0.0008)
15	-0.0022 (-0.0039, -0.0005)*	-0.0029 (-0.0052, -0.0005)*	-0.0010 (-0.0021, 0.0002)
16	-0.0012 (-0.0028, 0.0003)	-0.0006 (-0.0028, 0.0015)	-0.0004 (-0.0014, 0.0006)
17	-0.0014 (-0.0031, 0.0003)	-0.0016 (-0.0041, 0.0008)	-0.0010 (-0.0020, -0.0001)*
18	0.0005 (-0.0013, 0.0024)	0.0010 (-0.0015, 0.0035)	0.0005 (-0.0007, 0.0016)
19	-0.0022 (-0.0039, -0.0005)**	-0.0020 (-0.0043, 0.0004)	-0.0009 (-0.0020, 0.0003)
Stratified by standing height (cm)			
Q1 (132.9-160.2)	-0.0003 (-0.0013, 0.0007)	-0.0001 (-0.0016, 0.0014)	0.0004 (-0.0002, 0.0011)
Q2 (160.3-168.7)	-0.0009 (-0.0019, 0.0001)	0.0004 (-0.0010, 0.0017)	-0.0003 (-0.0009, 0.0003)
Q3 (168.8-190.9)	-0.0013 (-0.0023, -0.0004)**	-0.0012 (-0.0025, 0.0001)	-0.0009 (-0.0015, -0.0002)**
Stratified by race			
White people	-0.0017 (-0.0027, -0.0006)**	-0.0008 (-0.0023, 0.0007)	-0.0007 (-0.0014, -0.0000)*
Black people	-0.0003 (-0.0015, 0.0009)	0.0004 (-0.0013, 0.0021)	-0.0003 (-0.0010, 0.0004)
Mexican American	-0.0016 (-0.0030, -0.0002)*	-0.0023 (-0.0040, -0.0005)*	-0.0004 (-0.0013, 0.0005)
Other race	0.0018 (0.0007, 0.0030)**	0.0015 (-0.0000, 0.0030)	0.0010 (0.0002, 0.0017)*

All factors were adjusted. In the subgroup analysis, not adjusted for the stratification variable itself. *P < 0.05, **P < 0.01.

## 4 Discussion

We investigated the association between dietary FA and BMD using data from adolescents aged 12-19 in the NHANES dataset. This study ultimately comprised 3440 participants for data analysis. To assess the relationship between dietary FA and BMD at a deeper level and to make full use of these data, we stratified the total BMD according to quartiles. At the same time, we modeled the three FAs (SFA, MUFA, and PUFA) and BMD separately and performed subgroup analysis according to FA intake, age, gender, standing height, and race stratification to better assess the correlation between FA intake and BMD. Our analysis demonstrated that SFA was positively correlated with total BMD, left arm BMD, total BMC, and left arm BMC. Concurrently, MUFA intake was positively associated with BMC in several body regions, though its relationship with bone density did not achieve statistical significance. Importantly, PUFA intake was inversely correlated with BMD and BMC across most body areas. Subgroup analysis further revealed that variables such as age, sex, height, and ethnicity significantly impacted the relationship between dietary FA intake and BMD. In adolescents, significant variations in hormone levels influence bone growth and development. Families with higher economic status often have access to a healthier and more balanced diet, contributing to optimal nutritional status and improved bone health. Additionally, genetic factors play a significant role in bone development.

Short-chain fatty acids (SCFAs), which are saturated fatty acids comprising 1 to 6 carbon atoms, are generally associated with positive impacts on BMD. Lucas et al. (18) found that propionate or butyrate can protect bone health by regulating whole-body bone mass and preventing pathological bone loss. This phenomenon could be attributed to the suppression of gene expression, culminating in osteoclast differentiation and conferring protection against bone loss. Additionally, studies have indicated that adherence to the Mediterranean diet is linked to a reduced likelihood of experiencing fractures and an increased overall BMD. This association may be attributed, at least in part, to the production of short- chain fatty acids resulting from the fermentation of the diet’s abundant dietary fiber by intestinal microorganisms (24, 25). In another study, Carvalho et al. (23) found that diabetic mice fed a low-fat diet had a lower BMC than C57BL/6 mice fed a high-fat diet high in medium-chain fatty acids. These results support our research to some extent. In another study, the researchers assessed the risk of fractures by constructing a COX proportional hazards model combined with questionnaires, and the results showed that proper intake of PUFA and MUFA is beneficial to reduce the risk of total fractures (26). Macri et al. (27) studied the effects of high-MUFA diets on the bones of growing hypercholesterolemic rats and showed that replacing saturated fat with a high-MUFA diet improved bone mass and BMD. In a study from two-sample Mendelian Randomization, some MUFAs (such as palmitoleic acid, oleic acid, etc.) were positively associated with lower fracture risk, which seems to be the same conclusion as previous studies (28). However, in our study, it seems that inconsistent results have been obtained. In a specific population, the intake of MUFA has different effects on BMD in different parts. The specific mechanism must be explored further in the future. It is worth noting that there are generally positive effects between MUFA intake and BMC in different parts of the body, which appears to be consistent with previous research.

After reviewing a large amount of literature, it was found that the association between PUFA and bone health has garnered considerable attention among researchers, and the research conclusions are inconsistent or controversial. In a cross-sectional study from Spain, the results showed that serum Omega-3 levels were positively correlated with spine BMD and femoral neck BMD in postmenopausal women (29). Another Mendelian randomization (MR) analysis also found that alpha-linolenic acid and linoleic acid have a positive genetic causal relationship with estimated BMD and a negative genetic causal relationship with fracture risk (30). Nevertheless, the findings of a longitudinal study conducted over a period of 5 years revealed a negative correlation between increased intake of PUFA and BMD, specifically in the femoral neck region (31). Furthermore, Wang et al. (19) recently conducted a two-sample MR study, and the findings revealed a negative correlation between omega-6 PUFA and total body BMD. Notably, a systematic review and meta-analysis of randomized controlled trials showed that omega-3 PUFA supplementation may not significantly influence BMD and bone metabolism markers (32). However, they might offer potential short-term benefits for postmenopausal women’s health. It is evident that the conclusions of these studies are conflicting and controversial, partly because the researchers primarily investigated the association between overall BMD and FA, and insufficient sample sizes. Additionally, differences in race, sex, age, and other demographic factors introduced variability. Consequently, we utilized large-scale datasets to assess the correlation between PUFA and BMD, as well as BMC, across different body regions, accounting for potential confounders.

FA exhibits diverse roles in bone health. Research (33) indicates that SCFAs not only influence bone metabolism directly but also modulate immune and inflammatory responses, significantly enhancing bone formation. Specifically, butyrate has been shown to indirectly regulate Wnt10B, a key ligand in bone synthesis, via modulation of regulatory T cells, which suppress immune responses (34). Experimental studies reveal that butyrate, propionate, or acetate supplementation in drinking water increases bone mass in normal female mice and mitigates hormone-dependent bone loss in estrogen-deficient mice (18). Conversely, omega-6 PUFA adversely affects bone metabolism by inhibiting osteoblast genesis and promoting adipogenesis through mesenchymal stem cells (MSCs), mediated by alterations in the OPG/RANKL expression and PPARγ pathways (35, 36). Specifically, arachidonic acid and prostaglandin E2 exacerbate this effect by enhancing COX-2 expression, which leads to reduced osteogenesis (37). A lower Omega-6/Omega-3 PUFA ratio significantly improves bone density, highlighting the complex interactions of fatty acids with BMD (38). Understanding these intricate mechanisms of fatty acid metabolism and their impact on bone cell differentiation and homeostasis is essential for osteoporosis prevention and bone health preservation.

To our knowledge, this is the initial investigation into the association between fatty acid intake and BMD in adolescents. We examined the relationship of three specific FA with BMD using weighted multiple linear regression models. The analysis was stratified by gender, age, race, among other factors. Additionally, we explored the inherent correlations between FA and BMD. Crucially, the adequate sample size supports the development of strategic interventions for adolescent bone health. However, we acknowledge several limitations inherent in this research. Firstly, this is a cross-sectional study, which can only provide objective clinical evidence and cannot explain the intrinsic link between FA and BMD. Secondly, the investigation did not account for the current or recent use of medications by the participants, including but not limited to lipid-lowering agents and glucocorticoids, which are known to influence bone metabolism (39, 40). Finally, the NHANES database lacks data on specific FA classifications, including Omega-3 and Omega-6, precluding analysis of their individual relationships with BMD. Future studies should prioritize prospective, large-scale randomized controlled trials to establish robust, evidence-based conclusions regarding the effects of fatty acids on BMD. Moreover, the role of omega-3 PUFA in bone metabolism and health remains debated and warrants further investigation as a potential focal point in addressing clinical challenges.

In conclusion, our study reveals a significant positive correlation between the consumption of SFA and both total BMD as well as BMD in the left arm. In contrast, intake of PUFA demonstrated a significant negative correlation with these BMD indices. Notably, the association between MUFA consumption and BMD appeared to be influenced by variables such as specific body regions, age, gender, and ethnicity, yielding variable results. These findings underscore the intricate nature of bone metabolism. Based on our results, we recommend a balanced intake of dietary fatty acids among adolescents to optimize bone mass and ensure skeletal health.

## Data availability statement

Publicly available datasets were analyzed in this study. This data can be found here: www.cdc.gov/nchs/nhanes/.

## Ethics statement

The studies involving humans were approved by The National Center for Health Statistics’ Research Ethics Review Board. The studies were conducted in accordance with the local legislation and institutional requirements. Written informed consent for participation in this study was provided by the participants’ legal guardians/next of kin.

## Author contributions

Z-gW: Methodology, Supervision, Writing – original draft, Writing – review & editing. Z-bF: Software, Supervision, Writing – review & editing. X-LX: Funding acquisition, Supervision, Writing – review & editing.
